# Artificial intelligence and digital health for volume maintenance in hemodialysis patients

**DOI:** 10.1111/hdi.13033

**Published:** 2022-06-23

**Authors:** Vicki Sandys, Donal Sexton, Conall O'Seaghdha

**Affiliations:** ^1^ Royal College of Surgeons in Ireland Dublin Ireland; ^2^ St James's Hospital Dublin 8 Ireland; ^3^ Trinity Health Kidney Centre School of Medicine, Trinity College Dublin Dublin Ireland; ^4^ ADAPT: Research Centre for AI‐Driven Digital Content Technology Ireland

**Keywords:** hemodialysis, machine learning, volume, wearable sensors

## Abstract

Chronic fluid overload is associated with morbidity and mortality in hemodialysis patients. Optimizing the diagnosis and treatment of fluid overload remains a priority for the nephrology community. Although current methods of assessing fluid status, such as bioimpedance and lung ultrasound, have prognostic and diagnostic value, no single system or technique can be used to maintain euvolemia. The difficulty in maintaining and assessing fluid status led to a publication by the Kidney Health Initiative in 2019 aimed at fostering innovation in fluid management therapies. This review article focuses on the current limitations in our assessment of extracellular volume, and the novel technology and methods that can create a new paradigm for fluid management. The cardiology community has published research on multiparametric wearable devices that can create individualized predictions for heart failure events. In the future, similar wearable technology may be capable of tracking fluid changes during the interdialytic period and enabling behavioral change. Machine learning methods have shown promise in the prediction of volume‐related adverse events. Similar methods can be leveraged to create accurate, automated predictions of dry weight that can potentially be used to guide ultrafiltration targets and interdialytic weight gain goals.

## INTRODUCTION

Volume overload is prevalent among hemodialysis (HD) patients and is a key risk factor for all‐cause and cardiovascular mortality.^1^, ^2^ In the absence of residual renal function patients undergo a cyclical pattern of fluid accumulation between dialysis and fluid removal during dialysis. Chronic volume overload and interdialytic weight gain are key mechanisms underlying systemic and pulmonary hypertension, arterial stiffness, and left ventricular remodeling in this population.^3^, ^4^ In turn, excess rates of ultrafiltration required to correct volume overload can lead to complications associated with volume depletion, including myocardial stunning and cerebral ischemia.^5^, ^6^, ^7^

Assessing and maintaining fluid within a controlled, narrow range is difficult to achieve.^8^ No universal method of evaluating volume in HD patients exists,^8^ and standardized guidelines for adjusting dry weight are lacking. Newer tools such as bioimpedance, lung ultrasound, and relative blood volume can refine fluid status,^9^, ^10^ but the limitations of these methods are similar to that of the clinical exam; additional time and cost resources are needed to adequately assess and reassess a patient's fluid status and ensure that weight and ultrafiltration targets are tolerated.^8^, ^9^ To date, no method exists that can capture continuous, real‐time, interdialytic data in the outpatient setting and provide patients with feedback on their fluid status.^8^, ^11^

There are several emerging therapies that show promise.^12^, ^13^ Machine learning methods have been used to create prediction models for dry weight and volume‐related adverse events.^14^, ^15^ Wearable biosensors that evaluate extracellular volume and physiological parameters in heart failure may provide patients with actionable insights into their disease states.^14^ This review article provides an overview of

- Current methods of fluid assessment in HD patients
- Biosensors for fluid assessment in HD patients
- Uni‐ and multiparameter biosensors in heart failure
- Machine learning methods for the prediction of dry weight and volume‐related adverse events

### Current methods of fluid assessment in HD patients

Dry weight in HD is conceptualized as the euvolemic weight of the patient, or the lowest possible postdialysis weight achieved without minimal symptoms or signs of hypo or hypervolemia.^16^, ^17^ The parameters used to define dry weight, such as clinical signs, symptoms, weight, or blood pressure, can be insensitive in detecting changes in volume status.^9^, ^18^ Newer technologies, such as multifrequency bioimpedance and lung ultrasound, are useful in categorizing volume status and the use of these technologies may improve overall fluid control and blood pressure compared to the clinical exam.^19^, ^20^, ^21^, ^22^, ^23^A meta‐analysis has demonstrated that tool‐adjusted dry weight adjustment can improve left ventricular hypertrophy, cardiovascular events, and hospitalizations^21^; however, no improvement in all‐cause mortality has been demonstrated.^21^ Part of the issue in assessing the efficacy of these interventions may be the heterogeneity of device‐specific management protocols.^24^, ^25^, ^26^, ^27^ There is a lack of clear, device‐specific guidelines to set targets and guide dry weight adjustments.^10^, ^26^, ^28^ Bioimpedance outputs from the Fresenius body composition monitor (BCM), for example, still need to be contextualized, as excess ultrafiltration may not be tolerated by patients with autonomic dysfunction or low output heart failure.^7^

Clinician‐facing devices fail to address two barriers to achieving euvolemia: excess interdialytic weight gain and patients' tolerance of ultrafiltration. Without extending the duration of dialysis or increasing the number of treatments, nephrologists are limited in the amount of ultrafiltration that can be safely removed.^7^, ^28^ The Kidney Health Initiative published a report in 2019 on the need for innovation solutions to address fluid management, suggesting a multilevel approach that targets fluid assessment between and during dialysis sessions.^11^

### Biosensors for fluid assessment in HD patients

Most of the devices applicable for volume management in dialysis patients are designed for in‐center use, relying on physician and nurse interpretation of results.^11^ None of these methods provide patients with actionable insights into their fluid state, particularly between dialysis sessions.^8^ Advances in microelectronics have enabled the development of uniparameter and multiparameter wearable sensors capable of assessing volume^12^, ^13^, ^14^, ^15^ (Table 1). Only a few studies have addressed similar technology in dialysis patients (Table 1).

**TABLE 1:** Table of studies involving wearable impedance devices in heart failure and dialysis patients

Uniparameter heart failure wearable devices									
Authors	Year	Publication	*N*	Design	Follow‐up	Parameters	Patient interface	Method	Findings
Smeets et al.^29^	2020	JMIR Cardio	36	Prospective cohort study	12 months	Thoracic impedance, accelerometer	No	Wearable electrode‐based thoracic bioimpedance device. Measured twice a day for 3 days during hospitalization for acute decompensated HF	Thoracic impedance had a moderate negative correlation with fluid balance
Gyllensten et al.^30^	2016	JMIR Medical Informatics	91	Prospective cohort study	10 months	Thoracic impedance and weight scales	No	Wearable bioimpedance vest worn daily in the morning by chronic HF patients	Data were applied to three algorithms to predict impending HF hospitalizations. Impedance combined with trend algorithms improved the detection of impending heart failure events compared to weight
Darling et al.^31^	2017	JMIR Cardio	106	Prospective cohort study	75 days	Thoracic impedance	No	Wearable bioimpedance vest with textile electrodes worn daily for 5 min by patients with a recent admission for HF. Data were transmitted remotely via a mobile application over 45 days	Fifty‐seven patients transmitted sufficient data. Using an algorithm to assess thoracic impedance changes as a predictor of HF events, thoracic bioimpedance had 87% sensitivity, 70% specificity, and 72% accuracy
Multiparameter heart failure wearable devices									
Anand et al.^15^	2012	Journal of Cardiac Failure	543	Multicentre nonrandomized study	90 days	Thoracic impedance, electrocardiogram, and accelerometer	No	AVIVO Monitoring System: body worn adherent device used in patients with a recent heart failure admission	A total of 206 patients were used in the development, 337 in validation. In the validation cohort, the algorithm had 63% sensitivity, 92% specificity, and a false positive rate of 0.9 patient‐year in predicting heart failure events
Stehlik et al.^14^	2020	Circulation: Heart Failure	100	Multicentre nonrandomized study	3 months	Impedance, ECG, temperature, HR, HR variability, arrhythmia burden, and activity	Yes	Disposable multisensor patch paired with Bluetooth worn daily for 3 months in patients with a recent admission for heart failure. The personalized baseline model of physiological parameters was created from discharge data. Deviations from the baseline model were used to trigger a clinical event	The algorithm had a 76%–88% sensitivity and 85% specificity in detecting heart failure precursors
Khandwalla et al.^12^	2016	American College Cardiology Conference Poster	20	Prospective observational study	84 days	Thoracic impedance, stroke volume, cardiac output vital signs	No	toSense CoVa™ Monitoring System: Wearable thoracic impedance necklace is worn during dialysis. Thoracic fluid index, thoracic fluid volatility, and stroke volume were examined as predictors of heart failure events	Five events occurred (two heart failure hospitalizations, two outpatient heart failure exacerbations, and one episode of ventricular tachycardia/defibrillation). Thoracic fluid volatility ≥40% was able to predict 100% of events
Reljin et al.^32^	2020	Journal of Medical Internet Research medical informatics	43	Prospective observational study		Thoracic impedance, HR variability	No	Transthoracic impedance and ECG readings were taken with a wearable vest at admission, discharge, and in a control group without heart failure. Classification of patients into with fluid (baseline group) and patients without fluid (control and baseline group)	Cross‐validation accuracy of 92% using cubic support vector model of transthoracic impedance and heart rate variability
Dialysis wearable devices									
Schoutteten et al.^13^	2020	BioMed Central Nephrology	66	Prospective observational study	3 years	Thoracic impedance, accelerometer	No	Multifrequency wearable thoracic bioimpedance device using electrodes. ≥4 measurements taken intradialytically	Significant correlation (*r* = 0.755) between thoracic bioimpedance and ultrafiltration volume. Ultrafiltration volume prediction error variation of 300 ml using leave one out cross‐validation
Anand et al.^33^	2012	Congestive Heart Failure	25	Prospective nonrandomized trial	Two dialysis sessions and interdialytic days	Thoracic impedance, heart rate, posture, accelerometer	No	Normalized	Normalized bioimpedance correlated strongly with the volume of fluid removed (*r* = 0.98)
Segal et al.^12^	2016	American Society of Nephrology Conference Poster	72	Prospective observational study	≥1 dialysis session	Thoracic impedance, stroke volume, cardiac output, heart rate respiration rate, ECG waveforms	No	toSense CoVa monitoring system: thoracic impedance necklace worn during dialysis	Ultrafiltration volume correlated better with weight change predialysis and postdialysis rather than thoracic impedance

Thoracic impedance is the most commonly used technique. Rather than the multifrequency whole‐body impedance used by a standard dialysis body composition monitor (BCM),^27^ thoracic impedance is a segmental measurement of impedance that estimates intrathoracic fluid content.^34^ Thoracic impedance is more sensitive than weight in detecting changes related to fluid congestion but has several limitations. Measurements can be affected by body positioning, body composition, respiratory variations, and motion artifact and fluid distribution may not be uniform across the thorax.^34^, ^35^ As thoracic impedance is a compartmental approximation of fluid status, intrathoracic fluid might not reflect total body extracellular water content. Additionally, absolute impedance values, and the algorithms that analyze them, are not standardized across devices.^34^

Several thoracic impedance sensors have been evaluated in HD patients by comparing measurements with ultrafiltration volume and weight loss during dialysis.^12^, ^13^, ^33^ A wearable, electrode‐based, multifrequency thoracic impedance system validated against ultrafiltration volume removal is the most promising of these biosensors. In a study by Schoutteten et al., relative thoracic impedance resistance demonstrated a significant correlation with all ultrafiltration volumes categories (all *p* values < 0.01) in 66 patients, and a strong linear relationship was observed between the combination of relative thoracic impedance resistance and reactance and ultrafiltration volume at a frequency of 8 kHz (*R* ^2^ = 0.982 [CI: 0.912–0.993]). A cross‐validation approach used to predict the total fluid extraction at the last measurement point in dialysis showed a prediction error variation of 300 ml. The wearable nature of the design, and its predictive capacity, potentially opens up the possibility of personalized ultrafiltration feedback systems, although the impracticality of multiple electrodes may limit its use in ambulatory fluid monitoring.^13^

The CoVa monitoring system is an example of a multiparameter biosensor trialed in dialysis patients. The system consists of a wearable necklace applied before and during dialysis to measure heart rate (HR), respiratory rate (RR), thoracic impedance, stroke volume, and cardiac output, as well as ECG waveforms,^12^ with data sent wirelessly and recorded remotely. The impedance component has been validated against an FDA‐approved referenced device^36^ and tested in 72 dialysis patients during HD. In terms of tracking fluid changes, net fluid removed correlated better with intradialytic weight change than with thoracic impedance. However, a separate analysis in 33 HD patients has demonstrated a correlation between impedance measurements and fluid removed of *r* = 0.93 (*p* < 0.0001),^36^ suggesting that the system may be used in the future to assess longitudinal volume changes.

Prototypes of alternatives to thoracic impedance are being developed.^37^, ^38^ As extracellular water is disproportionately stored in the limbs due to high muscle content, calf bioimpedance represents a suitable location for segmental bioimpedance monitoring.^27^ Prototypes of patient‐facing devices that use either segmental calf bioimpedance or whole‐body, hand‐held bioimpedance measurements have been validated in bench studies but have yet to be tested prospectively in dialysis patients.^38^, ^39^

Lastly, research is ongoing into developing a consumer‐grade wearable capable of hydration status.^39^, ^40^ Miniaturized biosensors exploit physiological properties such as sodium, chloride, and potassium in sweat to approximate “hydration status,”^39^, ^41^ and several companies have developed wristwatches based upon light‐emitting diode (LED) technology which claim to track changes in interstitial fluid over time.^41^, ^42^ There is a paucity of published data on the accuracy and precision of commercial devices. With adaptation for the altered physiology of dialysis patients, such as decreases in sweat and fluctuations in skin sodium, consumer‐grade devices may be able to crudely assess fluid status during the interdialytic interval.^43^

### Biosensors in heart failure

Multiple noninvasive and invasive cardiac sensors exist that evaluate physiological changes preceding heart failure decompensation^14^, ^44^ (Table 1). There is comparatively more research on invasive biosensors; however, several noninvasive wearable electrodes and vests have been studied.^30^, ^31^, ^32^ Cardiac resynchronization devices and implantable cardioverter defibrillators (ICDs) have been redesigned to incorporate biosensors capable of assessing hemodynamic changes, heart rate derivatives, physical activity, thoracic impedance, and cardiac output.^45^ Although invasive sensors are unlikely to be implanted in a comorbid dialysis population,^46^ lessons can be learned from the remote monitoring systems that are used to integrate information from biosensors with clinical interfaces. The research performed on heart failure patients highlights the necessity of clear protocols to interpret values, the difficulty in obtaining usable data, and the need for multiparameter inputs to improve predictive accuracy.^31^, ^44^, ^47^, ^48^

### Remote monitoring in heart failure

Remote monitoring of hemodynamic changes in heart failure involves either physician monitoring or alert‐based strategies, in which alerts are generated once parameters cross a certain threshold. Neither has proven efficacy.^49^, ^50^ The Remote Management of Heart Failure using Implantable Electronic Devices (REM‐HF) trial, for example, randomized 1650 patients with heart failure and a variety of implantable devices to usual care versus active remote monitoring over 2.8 years and demonstrated no difference in the primary endpoint of mortality or unplanned hospitalization for cardiovascular causes (hazard ratio 1.01; 95% confidence interval [CI] 0.87–1.18; *p* = 0.87),^49^ despite high adherence and a low drop‐out rate of 4.8%. Strategies using automated text message alerts, rather than scheduled physician monitoring, have demonstrated a similar lack of benefit.^50^ In the Diagnostic Outcome Trial in Heart Failure (DOT‐HF) trial an audible alert strategy based upon implantable intrathoracic impedance devices did not reduce the primary end‐point composite of all‐cause mortality and heart failure hospitalizations (*p* = 0.063) after 14.5 months of follow‐up^50^ and drove an increase in hospitalization rates in the access arm (HR: 1.79 [95% CI: 1.08–2.95; *p* = 0.022]), as well as an increase in outpatient visits (250 vs. 84; *p* < 0.0001).^50^

The poor results seen using remote monitoring may be due to difficulty with the interpretation of outputs.^47^ Similar to bioimpedance, lung US, and relative blood volume (RBV) measurements, there is a lack of standardized guidelines for value interpretation.^10^, ^24^, ^25^, ^26^ Gyllensten et al. examined the impact of three alert algorithms on workload by creating a simulated home telemonitoring environment for heart failure using data from 91 patients chronic heart failure patients.^30^ Patients were asked to transmit bi‐daily weight, blood pressure, and bioimpedance data using a wearable vest. Alerts were created using three techniques: a weight rule of thumb algorithm defined as >2 kg increasing in weight over 3 days, a weight trend algorithm using moving‐average convergence divergence, and an impedance trend algorithm using a cumulative sum control chart. In this instance, trend algorithms improved the sensitivity to impending heart failure decompensations compared to trend algorithms using weight (sensitivity of weight‐moving average convergence divergence algorithm: 33%, sensitivity of noninvasive thoracic impedance‐cumulative sum control chart: 60%); however, the PPV remained low (10.9%).^30^ Data were reviewed remotely by healthcare professionals using a simulated interface and rated by importance. Raters were asked to decide on a theoretical action to be taken in response to the alert from several options. Agreement on actions between raters in response to the alerts was poor (kappa = 0.3), and up to 50% of bioimpedance alerts were disregarded. The authors hypothesized a lack of familiarity with impedance readings was a causal factor.^47^

If treatment targets are provided, outcomes are better. Direct wireless pulmonary artery pressure (PAP) devices, such as the FDA‐approved CardioMEMs, are based on the premise that PAP tends to rise before the clinical onset of heart failure symptoms. Data are sent to healthcare providers, accessed remotely, and subsequently interpreted. In the multicenter observational, single‐arm trial of 1200 patients with New York Heart Association class III heart failure, PAP declined significantly across patients, and the rate of hospitalization for heart failure was significantly lower at 1 year compared with the year before PAP device implantation (0.54 vs. 1.25 events/patient‐years, HR: 0.43 [95% CI: 0.39–0.47], *p* < 0.0001).^48^ In contrast to impedance‐based devices, target values were provided for PAP measurements, and management strategies were standardized.^48^

Tracking and interpreting biosensor data depend on the data being transmitted successfully. An observational study of an algorithm based on thoracic impedance changes from a prototype impedance vest showed an accuracy of 72% in predicting heart failure events in patients with a history of hospitalization for heart failure.^31^ However, usability was an issue. The vest was worn for 5 min each morning and paired wirelessly with a phone app, yet only half of the 180 consenting participants transmitted usable bioimpedance measurements on >65% of the study period despite study staff support.^31^

The efficacy of remote monitoring of hemodynamic parameters on outcomes in heart failure is uncertain.^49^, ^50^, ^51^, ^52^ However, there is a suggestion that similar to RBV and BCM measurements, output interpretation needs to be refined to optimize therapeutic responses.^48^

### Multiparameter biosensors in heart failure

Multiparameter models have demonstrated improved accuracy compared to single‐ and dual‐parameter devices in detecting heart failure events. Using an index of variables associated with heart failure events, such as HR, RR ratio to tidal volume, intrathoracic impedance, patient activity, and heart sounds, the multiSENSE trial created an algorithm with 70% sensitivity in detecting impending heart failure events, defined as heart failure admissions or unscheduled visits with intravenous treatment,^44^ compared to 21.6% sensitivity using intrathoracic impedance in the Detect Fluid Early from Intrathoraic Impedance Monitoring (Defeat‐PE) trial.^53^

Multiparameter prediction algorithms have also been used in noninvasive biosensors. The MUSIC and Link‐HF trials used wearable sensors to aggregate continuous data streams of physiological parameters and create predictive algorithms for heart failure events.^14^, ^15^ The Link‐HF trial involved a remote monitoring system consisting of a disposable sensor patch paired with Bluetooth to gather data relating to ECG, impedance, temperature, heart rate (HR), HR variability, arrhythmia burden, and activity. Personalized baseline models of vital signs patterns 72 h postdischarge were created, and the difference between observed and expected vital signs was refined into a single index called the multivariate charge index. If the daily average multivariate charge index reached a certain threshold, a clinical alert was triggered. Calculated ROC curves showed that the solution was able to detect precursors of hospitalization for heart failure exacerbation with 76%–88% sensitivity and 85% specificity, which was chosen for comparison with previous trials.^14^ The median time between the initial alert and readmission was approximately 6.5 (4.2–13.7) days, compared with 34 days in the MultiSENSE trial.^44^ The short median time between alert and hospitalization suggests that the algorithm provides information relating to the impending need for hospitalization, rather than providing a framework for the early detection of decompensation. However, this brief interval between the alert and the need for hospitalization is unlikely to prove an issue for timely intervention in dialysis patients attending the hospital two to three times a week. Such techniques, using noninvasive wearables to continuously measure volume status, combined with a comparison of changes in baseline physiological data and the generation of automated alerts, provide a potential framework for the prediction of volume‐related changes in dialysis patients.^14^, ^33^, ^44^

### Machine learning methods for the prediction of dry weight and volume‐related adverse events

Wearable sensors can potentially track fluid changes between and during dialysis by collecting data prospectively. In comparison, analyses of existing datasets may be able to refine treatments by predicting risks during dialysis and automating dry weight. Dialysis datasets are ideally suited to analysis using machine learning techniques. Large quantities of heterogeneous data are collected and stored at regular intervals during thrice‐weekly dialysis sessions, providing an optimal environment for machine learning to create predictive, individualized algorithms to guide clinical decisions.^54^, ^55^, ^56^

Machine learning methodologies have been employed for the detection of heart failure decompensations using existing data.^57^ Data mining techniques of existing health records have been used to analyze heart failure detection, severity estimation, prediction of readmission, and impending decompensation,^58^ using predictor features such as demographic and laboratory data, ECG tracings, echocardiogram findings, and physiological parameters, particularly heart rate variability (Table 2).^57^ ^,^ ^58^, ^62^, ^63^ There are fewer studies that target short‐term predictions of decompensation rather than a long‐term risk of hospitalization. Candelieri et al. explored the use of decision trees and support vector models in the prediction of heart failure decompensation, finding that a “hyper‐solution” framework encompassing an optimized ensemble of support vector models showed an accuracy of 87.35% and sensitivity of 90.91% in the early detection of heart failure decompensation.^60^, ^61^

**TABLE 2:** Table of studies using machine learning methods to predict heart failure decompensation

Prediction of impending heart failure decompensation								
Authors	Year	Publication	*N*	Data	Prediction target(s)	Method	Features	Outcome
Candelieri et al.^59^	2008	IEEE	49	Data collected every 2 weeks from patients 301 instances	Heart failure Decompensation within 2 weeks Risk No risk	Knowledge discovery process Decision trees List Support vector machine Radial basis function networks Leave‐one‐patient‐out‐approach	Systolic blood pressure (SBP) HR Respiratory rate (RR) Weight Temperature Total body water (TBW) Patient status: stable or decompensated Gender Age NYHA class Alcohol use Smoking	Decision trees best performance: Accuracy 92% Sensitivity 63% False‐positive rate of 6.9%
Candelieri et al.^60^	2009	IEEE	43	Data collected from patients every 2 weeks 301 instances	Heart failure decompensation within 2 weeks Risk No Risk	Decision Trees Support Vector Machine Leave‐one‐patient ‐approach Independent test set of 38 instances	SBP HR RR Weight Temperature TBW Patient status: stable or decompensated Gender Age NYHA class Alcohol use Smoking	Support vector machine performed best: Accuracy 97.3% Sensitivity 100%
Candelieri et al.^61^	2010	Open Medical Informatics Journal	50	Data collected from patients every 2 weeks 301 instances	Heart failure decompensation within 2 weeks Risk No risk	Hypersolution framework for Support vector machine Classification using genetic algorithm 10‐fold cross‐validation	SBP HR RR Weight Temperature TBW Patient status: stable or decompensated Gender Age NYHA class Alcohol use Smoking	Accuracy 87.35% Sensitivity 90.91% False‐positive 16.2%
Guidi et al.^62^	2014	IEEE	90	Retrospective data from a single‐center 136 records	Frequency of heart failure decompensation Heart failure severity Heart failure type Prognosis Short‐term and long‐term predictions of decompensation	Cross‐validation	Age Gender NYHA class Systolic blood pressure (SBP) Diastolic BP (DBP) Ejection Fraction BNP Heart rate (HR) ECG parameters	CART Accuracy: 87.60% Random Forests Accuracy: 85.60% CART algorithm chosen for transparency Short‐term and long‐term predictions of heart failure are not obtainable due to short follow‐up
Guidi et al.^63^	2015	BMC medical information decision making	250	Data collected from patients through scheduled outpatient visits, nurse visits every 1–2 weeks, blue‐tooth enabled home scales	Frequency of heart failure during the year after the first visit Classify heart failure as mild/moderate/severe	Integrated modules of: Decision support system Home‐based sensor devices Telemedicine infrastructure Random Forests 10‐fold cross‐validation	Height and weight SBP + DBP HR Oxygen saturation Ejection fraction BNP or NT pro‐BNP Bioelectrical impedance vector NYHA class 12‐lead ECG report Etiology Comorbidity Current therapy, pharmaceutical and surgical Nurse obtained features: qualitative parameters vital signs bioimpedance BNP	Frequency of decompensations within 1st year Accuracy 72% Sensitivity 60% Specificity 78% Heart failure Severity Accuracy 81% Sensitivity 76% Specificity 86%

Abbreviations: BNP: Brain natriuretic peptide; CART, Classification and Regression Tree; NYHA, New York Heart Association functional classification.

There has been significant progress in predicting complex volume‐related events during dialysis using machine learning^56^, ^64^, ^65^ (Table 3). Machine learning has been applied to the assessment of dry weight in dialysis patients using small dialysis datasets,^66^, ^67^, ^68^, ^69^, ^70^ as well in the prediction of volume‐related adverse events^56^, ^64^, ^65^ (Table 3). Barbieri et al. created a prototype algorithm for the prediction of intradialytic hypotension (IDH) using an artificial neural network consisting of 60 variables to create a multiple endpoint model predicting session‐specific Kt/V, fluid volume removal, HR, and blood pressure (BP).^64^ This was expanded upon in a seminal paper by Lee et al.^56^ Using 261,647 HD sessions with 1,600,531 time‐varying timestamps, the research group compared the ability of various machine learning models in predicting IDH, defined as a systolic BP < 90 mmHg, a decrease in systolic BP >20 mmHg or mean arterial pressure > 10 mmHg or prediction time BPs occurring within 1 h. The data were divided into training (70%), validation (5%), calibration (5%), and testing sets (20%). A recurrent neural network model was found to have superior performance in predicting the three definitions of IDH (AUC of 0.94 for prediction of systolic BP < 90 mmHg [CI: 0.94–0.94]) compared with the multilayer perceptron model, light gradient boosting machine, or logistic regression models. Both feature set ablation and feature‐ranking analyses were used to determine that time‐varying vital signs and HD settings contributed most to the model construction. Recurrent neural networks are particularly useful in managing the temporal information of a large dialysis dataset using time‐varying variables such as ultrafiltration and blood flow rate.^56^

**TABLE 3:** Overview of studies using machine learning methods to predict volume and volume‐related advice events in dialysis patients

Prediction of volume hemodialysis patients								
Study	Year	Publication	*N*	Data	Prediction target	Method	Features	Outcome
Guo et al.^66^	2021	Biomed Research International	476	Retrospective data from two centers >3 months on hemodialysis	Clinical dry weight as determined by the clinician	Sparse Laplacian‐ Random Vector functional Link Multiple Kernel Support Vector Regression Multi‐kernel Ridge regression Linear Regression Artificial Neural network Body Composition monitor (BCM) 10‐fold cross‐validation	Age Gender SBP DBP BMI HR Years on dialysis	Sparse Laplacian‐Random Vector functional Link performed best Mean diff: 0.09% of DW Standard deviation: 2.22 Limits of agreement: −4.27 to 4.44 No. out of agreement: 4.25% Root mean squared error (RMSE) 1.31 kg
Guo et al.^67^	2021	Frontiers in physiology	476	On hemodialysis	Clinical dry weight	Multiple Laplacian‐regularized Radial Basis Network Multiple Kernel SVR Multikernel Ridge regression Linear Regression Artificial Neural network BCM	Age Gender SBP DBP BMI HR Years on dialysis	Multiple Laplacian‐regularized Radial Basis Network performed best Mean difference: −0.04% of dry weight Standard deviation: 2.23% Limits of agreement −4.41‐4.33% No. out of agreement 3.57% RMSE 1.32 kg
Kim et al.^68^	2021	PLOS one	1672	Retrospective single center dataset >3 months on hemodialysis	Clinical overhydration (preweight‐ clinical dry weight)	Light Gradient boosting method Xtreme Gradient Boost Random forest methods Training 80% Testing 20% Prediction result verified with 20 random samples	Age Gender Body cell mass Hemoglobin Total protein Serum albumin Serum creatinine Phosphorus Serum sodium Serum potassium Adipose tissue mass Fat tissue index Height TBW Extracellular water Intracellular water Extracellular/Intracellular ratio Lean tissue index BMI Blood urea nitrogen Phosphate Calcium Chloride Diabetes mellitus Hypertension	Random Forest: Accuracy 39.36% Maximum error: 1.1 kg
Niel et al.^69^	2018	Pediatric Nephrology	14	Simulated dataset	Correction applied to nephrologist determined dry weight	Multilayer perceptron neural network Tested in 14 pediatric patients	Hydration status by bioimpedance Relative blood volume Postdialysis SBP	Mean difference between neural network determined dry weight and artificial intelligence determined dry weight = 0.497 kg (−1.33 to 1.29 kg)
Chiu et al.^70^	2005	American Journal of Nephrology	54	Hemodialysis	TBW measured by multifrequency body impedance analysis (BIA)	Artificial Neural network (ANN) Anthropometric data	Demographic data, anthropometric measurements, and multi‐frequency bioelectrical impedance analysis (MF‐BIA)	No difference between TBW‐ANN and TBW‐BIA (*p* = 0.639) Pearson's correlation coefficient (0.911) RMSE 2.48 kg
Prediction of volume‐related adverse events in hemodialysis patients								
Lee et al.^56^	2021	CJASN	9292	Retrospective single‐center dataset 261,647 hemodialysis sessions 1,600,531 independent timestamps Instances of IDH‐1 8% IDH‐2 31% IDH‐3 19%	Intradialytic hypotension 1: <90 mmHg within 1 h Intradialytic hypotension 2: decrease in SBP > 20 mmHg or decrease in mean arterial pressure > 10 mmhg within 1 h at initial time‐point Intradialytic hypotension 3: decrease in SBP > 20 mmHg or decrease in mean arterial pressure > 10 mmhg within 1 h at prediction time‐point	Recurrent neural network Multilayer perceptron model Light Gradient boosting machine model Logistic regression Training 70% Validation 5% Calibration 5% Testing: 20%	Age and sex Hemodialysis‐related features Vital signs Clinical information Laboratory findings Medications Details of features too innumerable to list	Recurrent neural network for Intradialytic hypotension‐1 Area under the receiver operating characteristic curve (AUCROC) 0.94 (95% confidence interval: 0.94 to 0.94) AUC precision‐recall curve for IDH‐1: 0.62 AUCROC IDH‐2: 0.87 AUCROC IDH‐3: 0.79
Barbieri et al.^64^	2019	Kidney Diseases		Retrospective single‐center dataset 766,000 records	Minimum SBP of the session Postdialysis HR Postdialysis weight Kt/V	Artificial neural network Training 70% Validation 10% Test 20%	Patient characteristics The historical record of physiological reactions Outcomes of previous dialysis sessions Pre‐dialysis data Prescribed dialysis dose for the index session	Minimum SBP of the session mean absolute error (MAE): 9.3 mmHg Post‐dialysis HR MAE: 7.3 bpm Post‐dialysis weight: MAE 0.23 kg KT/V MAE: 0.13
Chaudhuri et al.^65^	2021	BMC nephrology	616	Retrospective data from six centers 751,354 treatment records Instance of outcome 22%	Binary outcome of relative blood volume decreases at a rate of at least —6.5% per hour within the next 15 min	Extreme gradient boosting model Optical sensing device used to collect relative blood volume data 10‐s windows of data in the previous 15 min Training 80% Validation 10% Test data 10% Proof‐of‐concept dashboard developed for reporting predictions	Data from Optical Sensing device Hemodialysis machines Patient electronic health records 493 input variables	Precision: 0.33 Recall: 0.94 AUROC: 0.89

Abbreviations: ANN, artificial neural network; AUCROC, area under the receiver operating characteristic curve; BMI, body mass index; BIA: body impedance analysis; DBP, diastolic blood pressure; DW, dry weight; HR, heart rate; RBV, relative blood volume; RMSE, root mean squared error; SVR, support vector regression; SBP, systolic blood pressure; TBW, total body water.

Algorithms that predict ultrafiltration targets, that is, dry weight, have been less successful. Niel et al. created a preliminary multilayer perceptron model using simulated data and a limited number of input variables such as relative blood volume, bioimpedance, and blood pressure. The output was a correction factor applied to dry weight, and the model's real‐world performance was examined by assessing blood pressure responses following the application of the correction factor to the dry weights of 14 patients. The study's limited number of patients precludes firm conclusions on the success of the model.^69^

More conclusive studies have used retrospective HD data to create dry weight predictions. Gou et al. targeted clinical dry weight prediction using data from 476 HD patients augmented with 10 cross‐fold validation.^66^ A Sparse Laplacian regularized Random Vector Functional Link model consisting of seven input features (age, gender, systolic blood pressure, diastolic blood pressure, BMI, heart rate, and years on dialysis) was found to have the best performance in predicting clinical dry weight (*R* ^2^ = 0.9501, RMSE 1.3136 kg), in comparison to Multiple Kernel Support Vector Regression, Multikernel Ridge Regression, Linear Regression, an Artificial Neural Network, and bioimpedance using BCM.^66^ This work was built upon in a subsequent study that examined the use of a multiple Laplacian‐regularized radial basis function neural network model in the same cohort of 476 patients. The model had an excellent correlation with clinical dry weight (*R* = 0.95), and demonstrated a ratio‐out‐of‐agreement of <5% in a Bland–Altman analysis, suggesting that it could be interchanged with clinical dry weight. However, similar to the authors' previous study, the limits of agreement remained unacceptably wide for clinical applicability (−4.4% to 4.3% of dry weight).^67^ Both studies included a limited number of variables and did not consider time‐varying features that could have been used as volume indices. Lastly, Kim et al. were unable to predict clinical overhydration status accurately using machine learning methods in a retrospective dataset of 1672 patients. The prediction accuracy for clinical overhydration status, defined as the gap between the predialysis weight and clinical dry weight, was <40% using three different machine learning methods.^68^

## CONCLUSIONS

Current methods of assessing volume have diagnostic and prognostic value in assessing volume at a single point in time.^8^ While an accurate volume target is important to conceptualize goals and guide ultrafiltration, total ultrafiltration volume is limited by dialysis session length and patient tolerance of volume removal.^7^ Establishing an accurate dry weight lacks functionality if interdialytic volume gains are not minimized. At present, there is an absence of validated, interdialytic, wearable technology that can provide dialysis patients with the information and motivation to self‐manage.^11^

Advances in artificial intelligence, microelectronics, and the availability of large dialysis datasets have created an opportunity to develop individualized predictive algorithms.^54^ ^,^ ^56^, ^64^ The research performed by cardiologists into heart failure provides a roadmap for the development of continuous, predictive, personalized measurements of fluid changes combined with remote monitoring.^14^ The predictive parameters for volume overload in heart failure are similar to volume overload in dialysis patients, but the risks and environment in which those parameters occur differ. In comparison to outpatient heart failure monitoring, dialysis patients are exposed to nonphysiological stressors through intermittent hemodynamic instability precipitated by volume removal. The frequency and volume of HD data create an ideal environment for the training and testing of machine learning methods. The ability to use these data to predict hemodynamic responses on dialysis has been proven,^56^ and in the future, these predictions can be embedded with automatic responses to offset adverse outcomes like intradialytic hypotension. Synthesizing dialysis parameters with interdialytic volume metrics may similarly enable us to refine ultrafiltration goals, predict optimal dry weight, and assess and diagnose fluid states.

The use of artificial intelligence in dialysis is not without ethical and clinical limitations. A criticism of artificial intelligence, particularly artificial neural networks, is a lack of transparency on how predictive outputs are reached, in addition to challenges in generalizability beyond derivation datasets and issues with dataset shift. To maximize the accuracy of predictions there is a need for accurate, heterogeneous data inputs and external validation. Algorithms may lack clinical validity and utility, necessitating testing of real‐world applicability and interpretability. Lastly, optimizing the clinical status of a patient requires respecting their subjective experience; clinical signs, symptoms, and quality of life. The ideal use of artificial intelligence in fluid management at present is as a supplemental tool to aid patients and clinicians in making informed, reasoned decisions, based upon data‐driven outputs and clinical assessments.^71^

## FUNDING INFORMATION

Vicki Sandys and Conall O'Seaghdha are funded by DTIF grant DT 2019 0086. Donal Sexton is funded by Health Research Board grant HRB‐ARPP‐P‐2018‐011.

## CONFLICT OF INTEREST

The authors have no conflicts of interest to disclose.

## References

[1] Zoccali C , Moissl U , Chazot C , Mallamaci F , Tripepi G , Arkossy O , et al. Chronic fluid overload and mortality in ESRD. J Am Soc Nephrol. 2017;28:2491–7. 28473637 10.1681/ASN.2016121341 PMC5533242

[2] Siriopol D , Siriopol M , Stuard S , Voroneanu L , Wabel P , Moissl U , et al. An analysis of the impact of fluid overload and fluid depletion for all‐cause and cardiovascular mortality. Nephrol Dial Transplant. 2019;34:1385–93. 30624712 10.1093/ndt/gfy396

[3] Loutradis C , Sarafidis PA , Ferro CJ , Zoccali C . Volume overload in hemodialysis: diagnosis, cardiovascular consequences, and management. Nephrol Dial Transplant. 2020;36:2182–93. 10.1093/ndt/gfaa182 PMC8643589 33184659

[4] Pabst S , Hammerstingl C , Hundt F , Gerhardt T , Grohé C , Nickenig G , et al. Pulmonary hypertension in patients with chronic kidney disease on dialysis and without dialysis: results of the PEPPER‐study. PLoS One. 2012;7:e35310. 22530005 10.1371/journal.pone.0035310 PMC3329424

[5] Burton JO , Jefferies HJ , Selby NM , McIntyre CW . Hemodialysis‐induced cardiac injury: determinants and associated outcomes. Clin J Am Soc Nephrol. 2009;4:914–20. 19357245 10.2215/CJN.03900808 PMC2676185

[6] Shoji T , Tsubakihara Y , Fujii M , Imai E . Hemodialysis‐associated hypotension as an independent risk factor for two‐year mortality in hemodialysis patients. Kidney Int. 2004;66:1212–20. 15327420 10.1111/j.1523-1755.2004.00812.x

[7] Flythe JE , Kimmel SE , Brunelli SM . Rapid fluid removal during dialysis is associated with cardiovascular morbidity and mortality. Kidney Int. 2011;79:250–7. 20927040 10.1038/ki.2010.383 PMC3091945

[8] Flythe JE , Chang TI , Gallagher MP , Lindley E , Madero M , Sarafidis PA , et al. Blood pressure and volume management in dialysis: conclusions from a kidney disease: improving global outcomes (KDIGO) controversies conference. Kidney Int. 2020;97:861–76. 32278617 10.1016/j.kint.2020.01.046 PMC7215236

[9] Torino C , Gargani L , Sicari R , Letachowicz K , Ekart R , Fliser D , et al. The agreement between auscultation and lung ultrasound in hemodialysis patients: the LUST study. Clin J Am Soc Nephrol. 2016;11:2005–11. 27660305 10.2215/CJN.03890416 PMC5108194

[10] Moissl U , Arias‐Guillén M , Wabel P , Fontseré N , Carrera M , Campistol JM , et al. Bioimpedance‐guided fluid management in hemodialysis patients. Clin J Am Soc Nephrol. 2013;8:1575–82. 23949235 10.2215/CJN.12411212 PMC3805085

[11] Bonventre JV , Hurst FP , West M , Wu I , Roy‐Chaudhury P , Sheldon M . A technology roadmap for innovative approaches to kidney replacement therapies: a catalyst for change. Clin J Am Soc Nephrol. 2019;14:1539–47. 31562182 10.2215/CJN.02570319 PMC6777588

[12] toSense, Inc . Validation of Neck‐Worn Monitoring System ‐ A Study for Monitoring Subjects With Fluid‐Management Issues Using Repeated Measurements During Hemodialysis; 2014 https://clinicaltrials.gov/ct2/show/NCT02140905

[13] Schoutteten MK , Vranken J , Lee S , Smeets CJP , de Cannière H , van Hoof C , et al. Towards personalized fluid monitoring in haemodialysis patients: thoracic bioimpedance signal shows strong correlation with fluid changes, a cohort study. BMC Nephrol. 2020;21:264. 32652949 10.1186/s12882-020-01922-6 PMC7353684

[14] Stehlik J , Schmalfuss C , Bozkurt B , Nativi‐Nicolau J , Wohlfahrt P , Wegerich S , et al. Continuous wearable monitoring analytics predict heart failure hospitalization: the LINK‐HF multicenter study. Circ Heart Fail. 2020;13:e006513. 32093506 10.1161/CIRCHEARTFAILURE.119.006513

[15] Anand IS , Tang WH , Greenberg BH , Chakravarthy N , Libbus I , Katra RP , et al. Design and performance of a multisensor heart failure monitoring algorithm: results from the multisensor monitoring in congestive heart failure (MUSIC) study. J Card Fail. 2012;18:289–95. 22464769 10.1016/j.cardfail.2012.01.009

[16] Agarwal R , Weir MR . Dry‐weight: a concept revisited in an effort to avoid medication‐directed approaches for blood pressure control in hemodialysis patients. Clin J Am Soc Nephrol. 2010;5:1255–60. 20507951 10.2215/CJN.01760210 PMC2893058

[17] Sinha AD , Agarwal R . Setting the dry weight and its cardiovascular implications. Semin Dial. 2017;30:481–8. 28666069 10.1111/sdi.12624 PMC5668188

[18] Kraemer M , Rode C , Wizemann V . Detection limit of methods to assess fluid status changes in dialysis patients. Kidney Int. 2006;69:1609–20. 16501488 10.1038/sj.ki.5000286

[19] Machek P , Jirka T , Moissl U , Chamney P , Wabel P . Guided optimization of fluid status in haemodialysis patients. Nephrol Dial Transplant. 2010;25:538–44. 19793930 10.1093/ndt/gfp487 PMC2809248

[20] Zoccali C , Torino C , Mallamaci F , Sarafidis P , Papagianni A , Ekart R , et al. A randomized multicenter trial on a lung ultrasound–guided treatment strategy in patients on chronic hemodialysis with high cardiovascular risk. Kidney Int. 2021;100:1325–33. 34418415 10.1016/j.kint.2021.07.024

[21] Beaubien‐Souligny W , Kontar L , Blum D , Bouchard J , Denault AY , Wald R . Meta‐analysis of randomized controlled trials using tool‐assisted target weight adjustments in chronic dialysis patients. Kidney Int Rep. 2019;4:1426–34. 31701052 10.1016/j.ekir.2019.07.003 PMC6829199

[22] Hur E , Usta M , Toz H , Asci G , Wabel P , Kahvecioglu S , et al. Effect of fluid management guided by bioimpedance spectroscopy on cardiovascular parameters in hemodialysis patients: a randomized controlled trial. Am J Kidney Dis. 2013;61:957–65. 23415416 10.1053/j.ajkd.2012.12.017

[23] Loutradis C , Sarafidis PA , Ekart R , Papadopoulos C , Sachpekidis V , Alexandrou ME , et al. The effect of dry‐weight reduction guided by lung ultrasound on ambulatory blood pressure in hemodialysis patients: a randomized controlled trial. Kidney Int. 2019;95:1505–13. 31027889 10.1016/j.kint.2019.02.018

[24] Leung KCW , Quinn RR , Ravani P , Duff H , MacRae JM . Randomized crossover trial of blood volume monitoring–guided ultrafiltration biofeedback to reduce intradialytic hypotensive episodes with hemodialysis. Clin J Am Soc Nephrol. 2017;12:1831–40. 29018100 10.2215/CJN.01030117 PMC5672962

[25] Reddan DN , Szczech LA , Hasselblad V , Lowrie EG , Lindsay RM , Himmelfarb J , et al. Intradialytic blood volume monitoring in ambulatory hemodialysis patients: a randomized trial. J Am Soc Nephrol. 2005;16:2162–9. 15930095 10.1681/ASN.2004121053

[26] Chen H‐S , Lee KC , Cheng CT , Hou CC , Liou HH , Lin CJ , et al. Application of bioimpedance spectroscopy in Asian dialysis patients (ABISAD): serial follow‐up and dry weight evaluation. Clin Kidney J. 2013;6:29–34. 27818748 10.1093/ckj/sfs155 PMC5094401

[27] Davies SJ , Davenport A . The role of bioimpedance and biomarkers in helping to aid clinical decision‐making of volume assessments in dialysis patients. Kidney Int. 2014;86:489–96. 24918155 10.1038/ki.2014.207

[28] Agarwal R , Alborzi P , Satyan S , Light RP . Dry‐weight reduction in hypertensive hemodialysis patients (DRIP): a randomized controlled trial. Hypertension. 2009;53:500–7. 19153263 10.1161/HYPERTENSIONAHA.108.125674 PMC2679163

[29] Smeets CJP , Lee S , Groenendaal W , Squillace G , Vranken J , de Cannière H , et al. The added value of in‐hospital tracking of the efficacy of decongestion therapy and prognostic value of a wearable thoracic impedance sensor in acutely decompensated heart failure with volume overload: prospective cohort study. JMIR Cardio. 2020;4:e12141. 32186520 10.2196/12141 PMC7113802

[30] Cuba Gyllensten I , Bonomi AG , Goode KM , Reiter H , Habetha J , Amft O , et al. Early indication of decompensated heart failure in patients on home‐Telemonitoring: a comparison of prediction algorithms based on daily weight and noninvasive transthoracic bio‐impedance. JMIR Med Inform. 2016;4:e3. 10.2196/medinform.4842 PMC4777885 26892844

[31] Darling CE , Dovancescu S , Saczynski JS , Riistama J , Sert Kuniyoshi F , Rock J , et al. Bioimpedance‐based heart failure deterioration prediction using a prototype fluid accumulation vest‐Mobile phone dyad: an observational study. JMIR Cardio. 2017;1:e6057. 10.2196/cardio.6057 PMC6832026 31758769

[32] Reljin N , Posada‐Quintero HF , Eaton‐Robb C , Binici S , Ensom E , Ding E , et al. Machine learning model based on transthoracic bioimpedance and heart rate variability for lung fluid accumulation detection: prospective clinical study. JMIR Med Inform. 2020;8:e18715. 32852277 10.2196/18715 PMC7484776

[33] Anand IS , Doan AD , Ma KW , Toth JA , Geyen KJ , Otterness S , et al. Monitoring changes in fluid status with a wireless multisensor monitor: results from the fluid removal during adherent renal monitoring (FARM) study. Congest Heart Fail Greenwich Conn. 2012;18:32–6. 10.1111/j.1751-7133.2011.00271.x 22277175

[34] Tang WHW , Tong W . Measuring impedance in congestive heart failure: current options and clinical applications. Am Heart J. 2009;157:402–11. 19249408 10.1016/j.ahj.2008.10.016 PMC3058607

[35] Cuba‐Gyllensten I , Abtahi F , Bonomi AG , Lindecrantz K , Seoane F , Amft O . Removing respiratory artefacts from transthoracic bioimpedance spectroscopy measurements. J Phys Conf Ser. 2013;434:012018.

[36] toSense^TM^ . https://www.tosense.com/

[37] Ferreira J , Pau I , Lindecrantz K , Seoane F . A handheld and textile‐enabled bioimpedance system for ubiquitous body composition analysis. An initial functional validation. IEEE J Biomed Health Inform. 2017;21:1224–32. 28113962 10.1109/JBHI.2016.2628766

[38] Bonnet S , Bourgerette A , Gharbi S , Rubeck C , Arkouche W , Massot B , et al. Wearable impedance monitoring system for dialysis patients. Annu Int Conf IEEE Eng Med Biol Soc. 2016;5196–9. 28269435 10.1109/EMBC.2016.7591898

[39] One of the next Health Features that may be coming to Apple Watch relates to Tracking Hydration Levels of a user. *Patently Apple* . https://www.patentlyapple.com/patently‐apple/2021/08/one‐of‐the‐next‐health‐features‐that‐may‐be‐coming‐to‐apple‐watch‐relates‐to‐tracking‐hydration‐levels‐of‐a‐user.html

[40] Publications | Rockley Photonics. https://rockleyphotonics.com/publications/

[41] Seshadri DR , Li RT , Voos JE , Rowbottom JR , Alfes CM , Zorman CA , et al. Wearable sensors for monitoring the physiological and biochemical profile of the athlete. Npj Digit Med. 2019;2:1–16. 31341957 10.1038/s41746-019-0150-9 PMC6646404

[42] LVL – The First Wearable Hydration Monitor. *Kickstarter* . https://www.kickstarter.com/projects/lactate-threshold/lvl-the-first-wearable-hydration-monitor

[43] Qirjazi E , Salerno FR , Akbari A , Hur L , Penny J , Scholl T , et al. Tissue sodium concentrations in chronic kidney disease and dialysis patients by lower leg sodium‐23 magnetic resonance imaging. Nephrol Dial Transplant. 2021;36:1234–43. 10.1093/ndt/gfaa036 32252091

[44] Boehmer JP , Hariharan R , Devecchi FG , Smith AL , Molon G , Capucci A , et al. A multisensor algorithm predicts heart failure events in patients with implanted devices. JACC Heart Fail. 2017;5:216–25. 28254128 10.1016/j.jchf.2016.12.011

[45] Merchant FM , Dec GW , Singh JP . Implantable sensors for heart failure. Circ Arrhythm Electrophysiol. 2010;3:657–67. 21156777 10.1161/CIRCEP.110.959502

[46] Jukema JW , Timal RJ , Rotmans JI , Hensen LCR , Buiten MS , de Bie MK , et al. Prophylactic use of implantable cardioverter‐defibrillators in the prevention of sudden cardiac death in dialysis patients. Circulation. 2019;139:2628–38. 30882234 10.1161/CIRCULATIONAHA.119.039818

[47] Cuba Gyllensten I , Crundall‐Goode A , Aarts RM , Goode KM . Simulated case management of home telemonitoring to assess the impact of different alert algorithms on work‐load and clinical decisions. BMC Med Inform Decis Mak. 2017;17:11. 28095849 10.1186/s12911-016-0398-9 PMC5240411

[48] Shavelle DM , Desai AS , Abraham WT , Bourge RC , Raval N , Rathman LD , et al. Lower rates of heart failure and all‐cause hospitalizations during pulmonary artery pressure‐guided therapy for ambulatory heart failure. Circ Heart Fail. 2020;13:e006863. 32757642 10.1161/CIRCHEARTFAILURE.119.006863 PMC7434214

[49] Morgan JM , Kitt S , Gill J , McComb JM , Ng GA , Raftery J , et al. Remote management of heart failure using implantable electronic devices. Eur Heart J. 2017;38:2352–60. 28575235 10.1093/eurheartj/ehx227 PMC5837548

[50] van Veldhuisen DJ , Braunschweig F , Conraads V , Ford I , Cowie MR , Jondeau G , et al. Intrathoracic impedance monitoring, audible patient alerts, and outcome in patients with heart failure. Circulation. 2011;124:1719–26. 21931078 10.1161/CIRCULATIONAHA.111.043042

[51] Hindricks G , Taborsky M , Glikson M , Heinrich U , Schumacher B , Katz A , et al. Implant‐based multiparameter telemonitoring of patients with heart failure (IN‐TIME): a randomised controlled trial. Lancet. 2014;384:583–90. 25131977 10.1016/S0140-6736(14)61176-4

[52] McDonagh TA , Metra M , Adamo M , Gardner RS , Baumbach A , Böhm M , et al. 2021 ESC guidelines for the diagnosis and treatment of acute and chronic heart failure: developed by the task force for the diagnosis and treatment of acute and chronic heart failure of the European Society of Cardiology (ESC) with the special contribution of the heart failure association (HFA) of the ESC. Eur Heart J. 2021;42:3599–726.34447992

[53] Heist EK , Herre JM , Binkley PF , van Bakel A , Porterfield JG , Porterfield LM , et al. Analysis of different device‐based intrathoracic impedance vectors for detection of heart failure events (from the detect fluid early from intrathoracic impedance monitoring study). Am J Cardiol. 2014;114:1249–56. 25150135 10.1016/j.amjcard.2014.07.048

[54] Barbieri C , Molina M , Ponce P , Tothova M , Cattinelli I , Ion Titapiccolo J , et al. An international observational study suggests that artificial intelligence for clinical decision support optimizes anemia management in hemodialysis patients. Kidney Int. 2016;90:422–9. 27262365 10.1016/j.kint.2016.03.036

[55] Wang Y‐F , Hu TM , Wu CC , Yu FC , Fu CM , Lin SH , et al. Prediction of target range of intact parathyroid hormone in hemodialysis patients with artificial neural network. Comput Methods Prog Biomed. 2006;83:111–9. 10.1016/j.cmpb.2006.06.001 16839639

[56] Lee H , Yun D , Yoo J , Yoo K , Kim YC , Kim DK , et al. Deep learning model for real‐time prediction of intradialytic hypotension. Clin J Am Soc Nephrol. 2021;16:396–406. 33574056 10.2215/CJN.09280620 PMC8011016

[57] Tripoliti EE , Papadopoulos TG , Karanasiou GS , Naka KK , Fotiadis DI . Heart failure: diagnosis, severity estimation and prediction of adverse events through machine learning techniques. Comput Struct Biotechnol J. 2017;15:26–47. 27942354 10.1016/j.csbj.2016.11.001 PMC5133661

[58] Yasmin F . Artificial intelligence in the diagnosis and detection of heart failure: the past, present, and future. Rev Cardiovasc Med. 2021;22:1095–113. 34957756 10.31083/j.rcm2204121

[59] Candelieri A , Conforti D , Perticone F , Sciacqua A , Kawecka‐Jaszcz K , Styczkiewicz K . Early detection of decompensation conditions in heart failure patients by knowledge discovery: the HEARTFAID approaches. Comput Cardiol. 2008;893–6. https://doi/org/10.1109/CIC.2008.4749186

[60] Candelieri A , Conforti D , Sciacqua A , Perticone F . Knowledge discovery approaches for early detection of decompensation conditions in heart failure patients. Proceedings of the 2009 Ninth International Conference on Intelligent Systems Design and Applications. IEEE Computer Society; 2009. p. 357–62. 10.1109/ISDA.2009.204

[61] Candelieri A , Conforti D . A hyper‐solution framework for SVM classification: application for predicting destabilizations in chronic heart failure patients. Open Med Inform J. 2010;4:136–40. 21589851 10.2174/1874431101004010136 PMC3095094

[62] Guidi G , Pettenati MC , Melillo P , Iadanza E . A machine learning system to improve heart failure patient assistance. IEEE J Biomed Health Inform. 2014;18:1750–6. 25029521 10.1109/JBHI.2014.2337752

[63] Guidi G , Pollonini L , Dacso CC , Iadanza E . A multi‐layer monitoring system for clinical management of congestive heart failure. BMC Med Inform Decis Mak. 2015;15:S5. 10.1186/1472-6947-15-S3-S5 PMC4705509 26391638

[64] Barbieri C , Cattinelli I , Neri L , Mari F , Ramos R , Brancaccio D , et al. Development of an artificial intelligence model to guide the management of blood pressure, fluid volume, and dialysis dose in end‐stage kidney disease patients: proof of concept and first clinical assessment. Kidney Dis. 2019;5:28–33. 10.1159/000493479 PMC6388445 30815462

[65] Chaudhuri S , Han H , Monaghan C , Larkin J , Waguespack P , Shulman B , et al. Real‐time prediction of intradialytic relative blood volume: a proof‐of‐concept for integrated cloud computing infrastructure. BMC Nephrol. 2021;22:274. 34372809 10.1186/s12882-021-02481-0 PMC8351092

[66] Guo X , Zhou W , Lu Q , Du A , Cai Y , Ding Y . Assessing dry weight of hemodialysis patients via sparse Laplacian regularized RVFL neural network with L2,1‐norm. BioMed Res Int. 2021;2021:6627650. 33628794 10.1155/2021/6627650 PMC7880720

[67] Guo X , Zhou W , Yu Y , Cai Y , Zhang Y , Du A , et al. Multiple Laplacian regularized RBF neural network for assessing dry weight of patients with end‐stage renal disease. Front Physiol. 2021;12:2240. 10.3389/fphys.2021.790086 PMC8711098 34966294

[68] Kim HR , Bae HJ , Jeon JW , Ham YR , Na KR , Lee KW , et al. A novel approach to dry weight adjustments for dialysis patients using machine learning. PLoS ONE. 2021;16:e0250467. 33891656 10.1371/journal.pone.0250467 PMC8064601

[69] Niel O , Bastard P , Boussard C , Hogan J , Kwon T , Deschênes G . Artificial intelligence outperforms experienced nephrologists to assess dry weight in pediatric patients on chronic hemodialysis. Pediatr. Nephrol. 2018;33:1799–803. 29987454 10.1007/s00467-018-4015-2

[70] Chiu J‐S , Chong CF , Lin YF , Wu CC , Wang YF , Li YC . Applying an artificial neural network to predict total body water in hemodialysis patients. Am J Nephrol. 2005;25:507–13. 16155360 10.1159/000088279

[71] Loftus TJ , Shickel B , Ozrazgat‐Baslanti T , Ren Y , Glicksberg BS , Cao J , et al. Artificial intelligence‐enabled decision support in nephrology. Nat Rev Nephrol. 2022;1–14. 10.1038/s41581-022-00562-3 PMC9379375 35459850

